# The 35S U5 snRNP Is Generated from the Activated Spliceosome during *In vitro* Splicing

**DOI:** 10.1371/journal.pone.0128430

**Published:** 2015-05-28

**Authors:** Olga V. Makarova, Evgeny M. Makarov

**Affiliations:** 1 Department of Cellular Biochemistry, Max Planck Institute for Biophysical Chemistry, Goettingen D-, Germany; 2 Department of Biochemistry, University of Leicester, Leicester, United Kingdom; 3 Division of Biosciences, College of Health and Life Sciences, Brunel University London, Uxbridge, United Kingdom; CNRS UMR7275, FRANCE

## Abstract

Primary gene transcripts of eukaryotes contain introns, which are removed during processing by splicing machinery. Biochemical studies *In vitro* have identified a specific pathway in which introns are recognised and spliced out. This occurs by progressive formation of spliceosomal complexes designated as E, A, B, and C. The composition and structure of these spliceosomal conformations have been characterised in many detail. In contrast, transitions between the complexes and the intermediates of these reactions are currently less clear. We have previously isolated a novel 35S U5 snRNP from HeLa nuclear extracts. The protein composition of this particle differed from the canonical 20S U5 snRNPs but was remarkably similar to the activated B* spliceosomes. Based on this observation we have proposed a hypothesis that 35S U5 snRNPs represent a dissociation product of the spliceosome after both transesterification reactions are completed. Here we provide experimental evidence that 35S U5 snRNPs are generated from the activated B* spliceosomes during *In vitro* splicing.

## Introduction

The majority of protein coding genes in eukaryotes are interrupted by introns, which are removed from mRNA precursors (pre-mRNA) via a process of pre-mRNA splicing to produce mature mRNA for protein translation. Intron removal and joining of exons is carried out by the spliceosome which catalyses two sequential *trans*-esterification reactions with single nucleotide precision to generate a correct message. The spliceosome is a very complex and dynamic cellular machine comprising five small nuclear (sn) RNAs (U1, U2, U4, U5, U6) and many, but still an uncertain number of proteins, around 200, which interact with pre-mRNA in a temporally ordered manner (reviewed in [1]). The spliceosome assembly is initiated by the recognition of the 5’ splice site (ss) by the U1 snRNP and the 3’ss by the protein factors SF1/mBBP and U2AF to form the Early (E) complex. In the next step, the pre-spliceosomal complex A is formed upon the ATP-dependent association of the U2 snRNP with the branch site. The fully assembled spliceosome contains the U4/U6.U5 tri-snRNP particle in addition, and it is designated as the pre-catalytic B complex. Next, the PRP19/CDC5L complex is recruited and the spliceosome undergo dramatic conformational changes resulting in dissociation of the U1 and U4 snRNPs and establishing a complex network of RNA-RNA and RNA-protein interactions. The first transesterification reaction results in formation of complex C with the 5' exon detached and intron-lariat with the 3' exon. The second transesterification reaction results in joining of exons and displacement of intron-lariat. After this, the spliceosome is subjected to disassembling rearrangements in order to release mRNA and re-cycle the snRNPs.

Using different purification techniques a number of the spliceosomal complexes have been isolated from HeLa nuclear extracts and their protein compositions and 3D-structures were determined by mass-spectrometry (MS) and cryo-electron microscopy, respectively. The inventory list includes the spliceosomal E complex [2], complex A [3], complex B [4], complex B^act^ [5], complex BΔU1 [6], complex B* [7] and complex C [8]. A comparison of these spliceosomal complexes revealed dramatic changes of their protein composition during spliceosome assembly (reviewed in [9]). The counterpart complexes (B, B^act^ and C) were purified from yeast and analysis of their protein composition revealed similar dynamics of proteins exchange during spliceosome activation [10]. Moreover, the architecture of the counterpart spliceosomal complexes as studied by electron microscopy appeared to be very similar, which is in support of the idea that the overall mechanism of pre-mRNA splicing is evolutionary conserved [5,10].

Using immunoaffinity selection with antibodies against the SKIP protein, we previously isolated the human 45S complex, named the activated spliceosome or complex B* [7]. This complex contained the pre-mRNA, the U2, U5 and U6 snRNAs (no U1 or U4 snRNAs) and was able to catalyse both *trans*-esterification reactions when supplemented with proteins from the micrococcal nuclease (MN) treated nuclear extract [7]. Consistent with the RNA composition, the proteins associated with U1 and U4 were not identified by MS in the B* complex whereas the U2- and U5-associated proteins remained associated. Importantly, the B* complex also contained fifteen proteins of the PRP19/CDC5L complex and its related proteins.

Unexpectedly, the same anti-SKIP antibodies precipitated the novel 35S U5 snRNP from HeLa nuclear extract. Comparison of the protein compositions of the 35S U5 snRNP and the B* complex revealed that most proteins of the 35S U5 particle are components of the B* complexes, including the PRP19/CDC5L and related proteins. This observation has suggested that the 35S particle is either a precursor or product of the activated spliceosome. A number of evidences favour the latter suggestion. First, the 35S U5 snRNP particle contains a considerably larger number of proteins compared to the earlier purified 20S U5 particle, but its three proteins, namely, 15K, 52K and 100K, were not detected either in the B* complex or in the 35S snRNP, indicating that they have already dissociated at the previous stages of spliceosome assembly. Second, at least two 35S U5 proteins, the second step factor hPrp17 and the RNA helicase DDX35 were not detected in the B* complex, but they were found in the C complex, suggesting that these proteins stably associate with the 35S U5 snRNP after spliceosome activation.

To test the hypothesis that 35S U5 snRNPs are in fact remodelled post-spliceosomal U5 snRNP particles we have devised the solid phase splicing assay in which the purified B* complexes were kept attached to the beads via anti-SF3a66K antibodies and incubated under conditions allowing splicing to occur in the absence of endogenous snRNPs. We have observed the dissociation of U5 snRNA from the beads during *in vitro* splicing and provided evidence that the U5 snRNA is released in a form of the 35S particle, confirming that the 35S snRNP is a product generated from the B* complex.

## Materials and Methods

### In vitro splicing and antibody reagents

The HeLa nuclear extract was prepared according to Dignam *et al*. [11]. The micrococcal nuclease (MN) treated extract used for splicing was obtained by incubating the HeLa nuclear extract in D buffer containing 1.5 mM CaCl_2_ and 0.5 units/μL micrococcal nuclease (Amersham) for 5 min at 30°C. The nuclease activity was quenched by addition of EGTA to 4.5 mM followed by incubation for 1 min at 30°C. The digestion of snRNAs was analysed by denaturing gel electrophoresis followed by silver staining.

All the antibodies were raised against the customer peptides in rabbits by Eurogentec according to the manufacturer licenses and policies, and were affinity purified using a SulfoLink column (Pierce) containing the cognate peptide. Antibodies were raised against a peptide of splicing factors SKIP (aa 1470–1485), SF3a66K (aa 516–531), and DDX35 (aa 392–409), and were described previously in [7], [12], and [8], respectively.

### Double affinity purification of activated spliceosomes

A 2.4-mL splicing reaction containing the 40% HeLa nuclear extract and 10 nM ^32^P-labelled MINX pre-mRNA (40000 cpm/pmol) was incubated at 30°C for 10 min. Heparin was added to a final concentration of 0.5 mg/mL and incubation continued for 5 min at 30°C. The following steps were carried out at 4°C. Aliquots of 0.5 mL and 0.3 mL (bed volume) of protein A-sepharose (PAS), pre-blocked with 0.5 mg/ml of BSA and 50 μg/mL of yeast tRNA, were charged with 250 μg of the affinity purified anti-SKIP antibodies and 140 μg of anti-SF3a66K antibodies, respectively. The splicing reaction was diluted 6 fold with IP150 buffer (20 mM HEPES, pH 7.9, 150 mM NaCl, 1.5 mM MgCl_2_, 0.5 mM DTT, 0.05% NP-40), and incubated for 2 hours with 0.5 mL of PAS charged with the anti-SKIP antibodies. Beads were washed 5 times with IP150 buffer, and the bound material was eluted by incubating for 1 hour with 2 mL IP150 buffer containing 5% glycerol and 0.6 mg/mL cognate peptide. The eluate was then incubated with 0.3 mL of PAS charged with the anti-SF3a66K antibodies for 1 hour and the beads were washed 5 times with IP150 buffer, twice with IP150 buffer containing 150 mM KCl instead of NaCl, and twice with IP buffer containing 50 mM KCl. The spliceosomal complexes were kept bound to beads and stored on ice.

### Solid phase splicing assay

The activated spliceosomes immobilised on PAS via anti-SF3a66K antibodies were supplemented with 20% of MN-treated nuclear extract and incubated for 80 min either under splicing conditions: in the presence of 2 mM of ATP and at 30°C; or at 0°C; or in the absence of ATP at 30°C. At the end of the incubation, heparin was added to the final concentration of 0.5 mg/mL, and incubation continued for 5 min at 30°C. After that, the supernatant was separated from the beads and placed on ice. Aliquots (20 μL) of the supernatant from each reaction were taken for radioactivity measurements by Cherenkov counting. To analyse the complexes, a 0.2 mL aliquot of the supernatant was loaded onto each 4.0 mL linear 10–30% glycerol gradient prepared with IP150 buffer without NP-40, and centrifuged in a TH660 rotor (Sorvall) for 14 hours at 24,500 rpm. Gradients were fractionated into 175 μL aliquots and RNA was extracted from each fraction. An aliquot of RNA from each fraction was separated by 14% denaturing PAGE and transferred to the Hybond N membrane. The ^32^P-labeled RNAs originated from the pre-mRNA were detected directly by autoradiography, whereas the U5 snRNAs were visualised by Northern blotting with the U5-specific probe as described in [7]. The membrane was exposed to a PhosphorImager (Molecular Dynamics) before and after Northern hybridisation and signals were quantified using ImageQuant software.

### Analytical immunoprecipitation

An aliquot of 60 μL PAS beads, pre-blocked with 0.5 mg/mL of BSA and 50 μg/mL of yeast tRNA, was charged with 30 μg of the affinity purified anti-DDX35 antibodies. A half of the DDX35-containing beads was blocked with cognate peptide by incubation for 1 hour with the peptide at the concentration of 0.6 mg/mL followed by 3 washes with IP150 buffer to remove unbound peptide. The gradient fractions, corresponding to the 35S (9–12) or 45S (14–17) region, were pulled, diluted 5 times with IP150 buffer and used as input material. A 1.4 mL aliquot of indicated fractions was incubated for 1 h at 4°C with 15 μL of PAS-DDX35 beads which were either pre-blocked with the cognate peptide or not. The beads were then washed 5 times with IP150 buffer and co-precipitated RNA was recovered by phenol-chloroform extraction, 3’ end labelled with ^32^P-pCp and analysed by 10% denaturing PAGE followed by autoradiography as described previously [13].

## Results and Discussion

**Fig 1:**
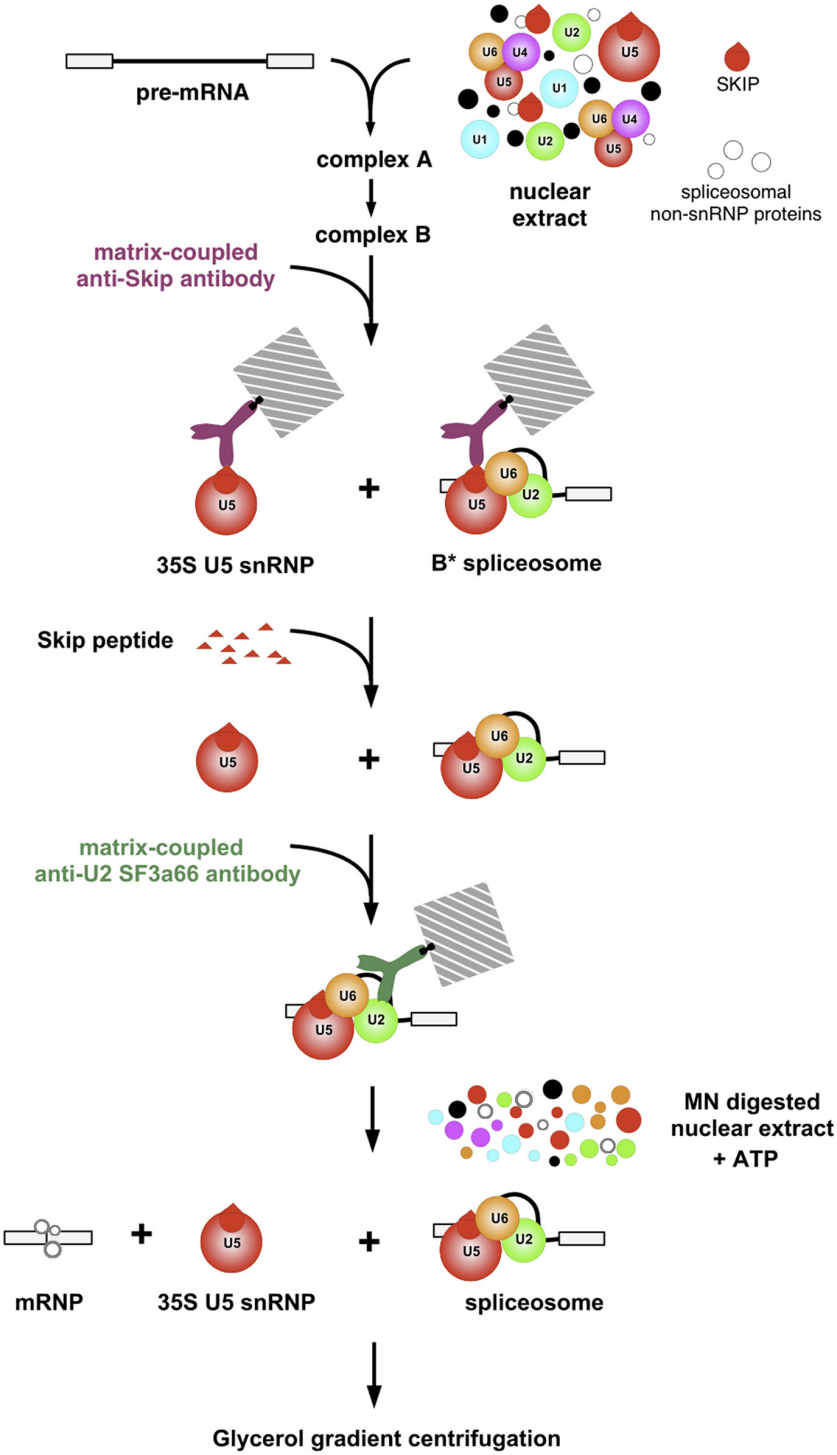
Flowchart of the procedure designed for detecting the release of 35S U5 snRNPs from the B* complexes during in vitro splicing. Splicing complexes assembled on the radioactively labelled pre-mRNA were precipitated with the matrix-coupled anti-SKIP antibodies, and eluted by competition with the cognate peptide. Eluted complexes were subjected to the second immunoprecipitation with the beads coupled with anti-SF3a66K antibodies. The complexes bound to the beads were incubated under splicing conditions in the presence of MN-treated HeLa nuclear extract. The complexes released into the supernatant were analysed by gradient density centrifugation.

The conventional in vitro splicing assay cannot be effectively used to address the product-precursor relation of the 35S U5 snRNP and the B* complexes as a HeLa nuclear extract contains endogenous 35S U5 particles which would interfere with the detection of complexes generated during the course of splicing. To overcome this obstacle, we have devised the solid phase splicing assay, shown schematically in Fig 1, in which the purified B* complexes were kept attached to the beads via the antibodies and incubated under conditions allowing splicing to occur in the absence of endogenous snRNPs. The B* spliceosomes were assembled in nuclear extracts on the radioactively labelled pre-mRNA and isolated using antibody against the SKIP protein, which is also a component of the endogenous 35S U snRNP. To select the B* complexes from the mixture, the complexes were first eluted by competition with the SKIP peptide and then, the B* spliceosomes were selected using antibodies against the U2 snRNP-specific protein SF3a66K, which is not present in the 35S U5 snRNP. The double-affinity purified B* complexes, eluted from the beads by competition with the SF3a66K antigenic peptide, were structurally intact and migrated though the density gradient as the 45S complex [7]. These complexes are considered to represent the stable core of the activated spliceosome and while being isolated under stringent conditions, in the presence of heparin, require additional factors for pre-mRNA processing. Previously, we have shown that the activated spliceosomes eluted from beads were catalytically active and spliced pre-mRNA in the presence of micrococcal nuclease (MN)-treated extract [7]. In this study, we kept the double-affinity purified B* complexes bound to the beads and allowed them to splice by supplementing with MN-treated nuclear extract.

**Fig 2:**
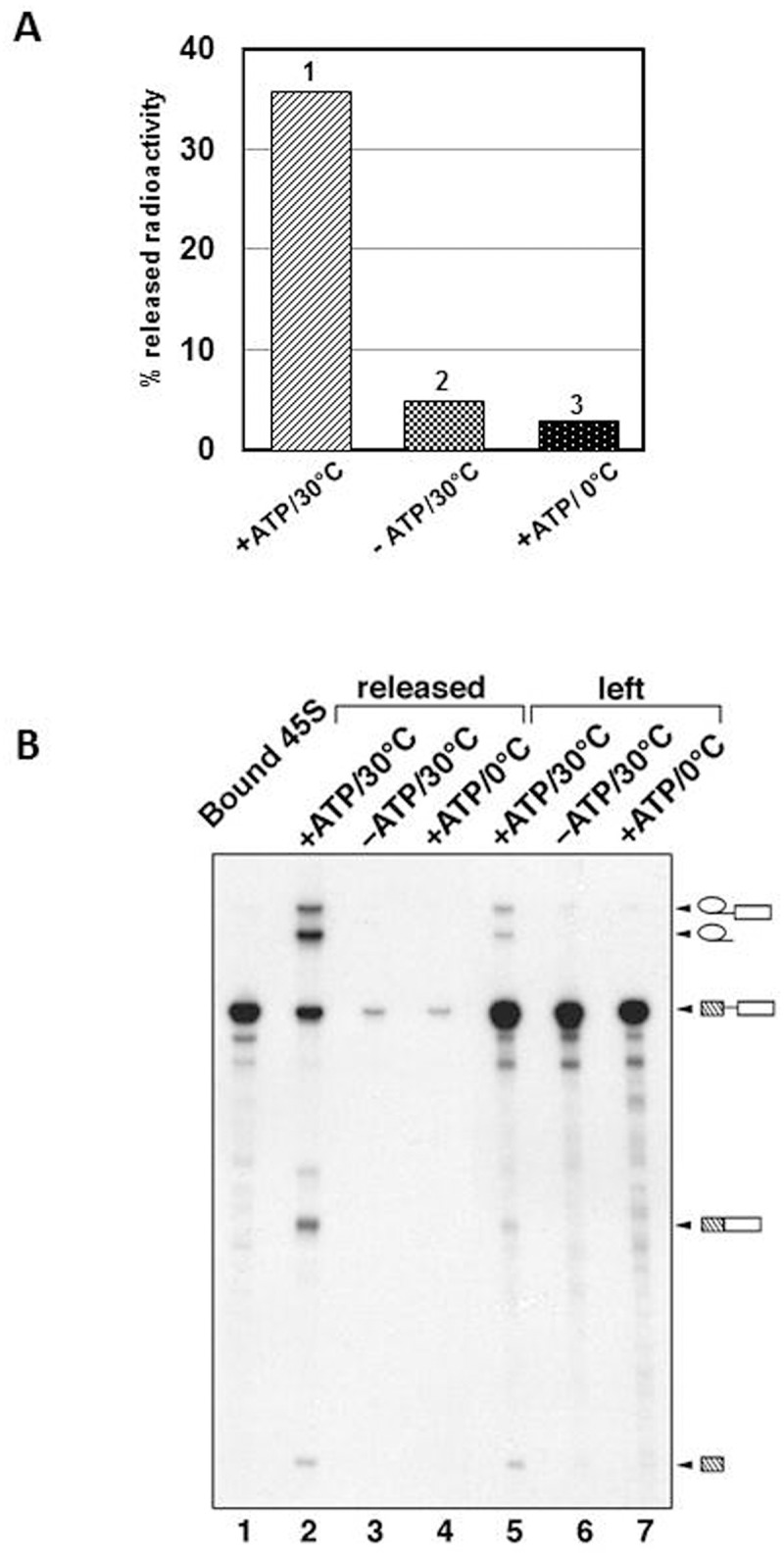
The activated spliceosomes immobilised to the beads are capable of catalysing both *trans*-esterification reactions in the presence of MN-treated nuclear extract. (**A**) The Cherenkov counting analysis of the material released from the beads under splicing condition (1—in the presence of ATP at 30°C) and under conditions, which do not support splicing (2—in the absence of ATP at 30°C; 3—in the presence of ATP at 0°C). (**B**) The RNA extracted from both the supernatants (lane 2, 3, 4) and the beads (lane 1, 5, 6, 7) after incubation under splicing conditions (lane 2 and 5) and under conditions which do not support splicing (lane 3, 4 and 6, 7), was analysed by denaturing PAGE followed by autoradiography. Lane 1 contains the RNA bound to the beads prior to the incubation in the presence of MN-treated nuclear extract. Identities of the RNA species are shown on the right.

We first checked the supernatant of the reaction for the presence of splice products. Fig 2A shows a diagram of Cherenkov counting of the supernatants of the reactions carried out under splicing conditions (+ATP, 30°C) or at 0°C or in the absence of ATP. A significant amount of radioactivity was released to the supernatant when the complexes were incubated at 30°C. The analysis of RNA content of the reactions' supernatants (Fig 2B), revealed that the B* complexes incubated under splicing permissive conditions (+ATP, 30°C), were capable to splice and produced the spliced product and intermediates (lane 2) whereas the supernatants of the control reactions (no ATP or 0°C, lanes 3–4) contained only a minor amount of unprocessed pre-mRNA (compare lanes 6–7 and 3–4), which was dissociated from the beads without processing. The analysis of material attached to the beads has confirmed that complexes incubated under non-permissive splicing conditions were left intact. Thus, we have demonstrated that the activated spliceosomes attached to the beads via anti-SF3a66K antibodies were able to carry out both steps of splicing when supplemented with protein splicing factors.

**Fig 3:**
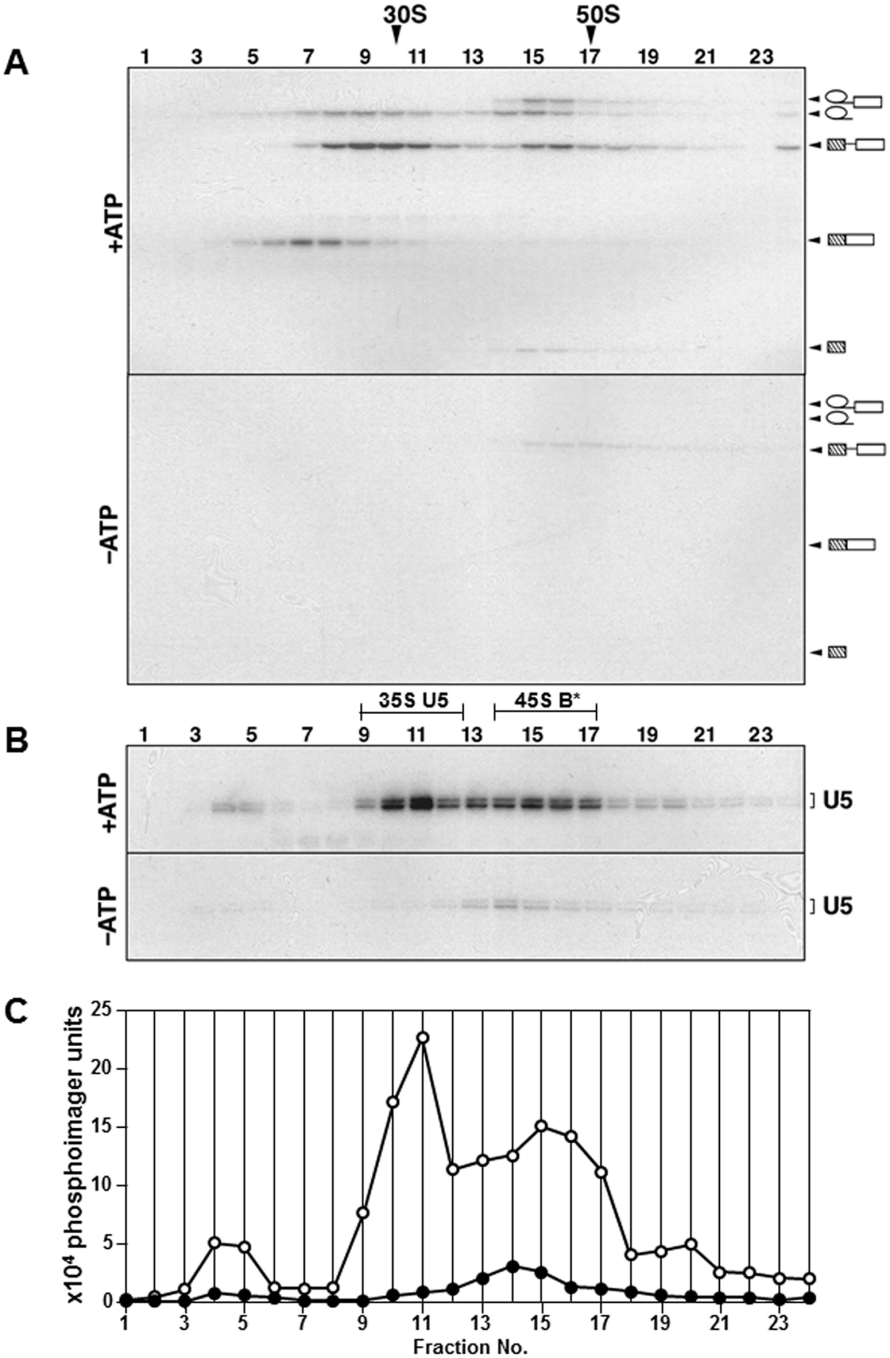
Characterisation of the complexes released into the supernatant from immobilised activated spliceosomes during splicing in the presence or absence of ATP by glycerol gradient centrifugation. (**A**) The RNA extracted from each gradient fraction was fractionated by denaturing PAGE and the ^32^P-containing species were detected by autoradiography. S-values were determined by comparison with the reference gradients containing 30S and 50S ribosomal subunits. The RNA identities are shown on the right. (**B**) Northern blot analysis of gradient fractions from panel A with the U5 snRNA specific probe. Fractions corresponding to the 35S U5 snRNP and 45S B* spliceosomes are underlined. (**C**) Quantification of the signals corresponding to the U5 snRNA (panel B) using a PhosphorImager. Open circles correspond to the reaction carried out in the presence of ATP and closed circles—in the absence of ATP.

For the analysis of complexes present in the supernatant of reactions we fractionated them using glycerol density centrifugation. The RNA from the gradient fractions was extracted, separated by denaturing PAGE and visualised by autoradiography (Fig 3A). The supernatant of the reaction carried out under splicing conditions (+ATP, 30°C) exhibited a distinct sedimentation profile separating several complexes, whereas the supernatants from the control reactions (either incubated at 0°C in the presence of ATP or at 30°C in the absence of ATP) contained only a trace amount of the pre-mRNA migrating in the 45S region of the gradient corresponding to the dissociated unprocessed spliceosomes. The splicing products detected on the sedimentation profile of active splicing reaction are: spliced mRNA (fractions 5–9), cleaved lariat (fractions 8–11), and the spliceosomes that undergone the first trans-esterification reaction and contain first exon and intron-lariat with the second exon (fractions 14–17). Small amount of the B* complex containing unprocessed pre-mRNA was present in fractions 14–17, corresponding to the 45S region of the gradient. Fractions 8-11contained unspliced pre-mRNA but the nature of these complexes was not determined (25-30S).

Since a portion of pre-mRNA was processed completely and produced mRNA, we analysed the presence of the post-spliceosomal 35S U5 snRNP by looking at the distribution of U5 snRNA using northern blotting (Fig 3B). The RNA samples from the gradient fractions was separated by denaturing PAGE, transferred to a nylon membrane, and hybridised with the probe specific to the U5 snRNA. We observed a strong signal in fractions 10–11 that matches the complexes sedimenting with the 35S value. The residual signal was also detected in fractions 14–17 that correspond to the B* spliceosomes and complexes that undergone first transesterification reaction and therefore contain U5 snRNA. The quantification of the U5 snRNA signal by phosphorimager (Fig 3C) identified that more than 50% of the U5 snRNA were present in the 35S region of the gradient. In the gradient of the control reaction carried out in the absence of ATP no U5 signal was detected in the corresponding region. This also confirmed the absence of 20S U5 snRNP in the MN-treated nuclear extract.

**Fig 4:**
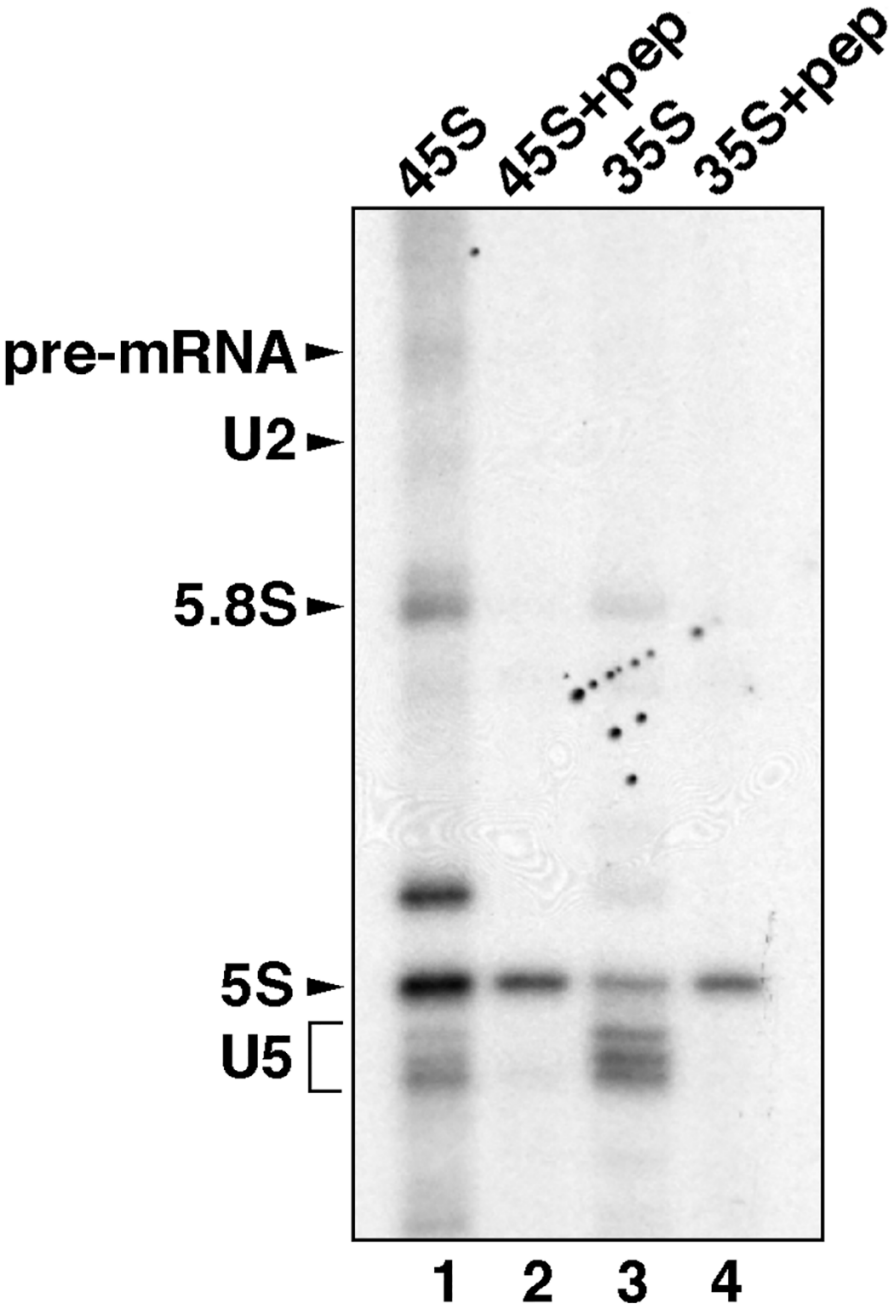
Immunoprecipitation of the gradient purified 45S and 35S complexes using anti-DDX35 antibodies. The fractions from the gradient analysed in Fig 3, corresponding to the 35S region (fractions 9–12—lane 3 and 4) or the 45S region (fractions 14–17—lane 1 and 2), were combined and subjected to immunoprecipitation with the anti-DDX35 antibodies (lane 1 and 3) or with the anti-DDX35 antibodies pre-blocked with antigenic peptide (lanes 2, 4). The RNA, extracted from the beads, was labelled with ^32^P-pCp and analysed by denaturing PAGE followed by autoradiography. Identities of the RNA species are shown on the left. The bands marked 5.8S and 5S correspond to rRNA that was precipitated unspecifically.

To confirm the identity of the 35S U5 snRNPs released during splicing we used antibody to the DDX35 protein to precipitate 35S particles from the gradient fractions. The DEAD box RNA helicase DDX35 was identified as a component of the 35S U5 snRNPs and the C complex but not activated spliceosomes isolated either under stringent, in the presence of heparin, (B*) or physiological (B^act^) conditions [7, 14]. The immunoprecipitations were carried out from combined fractions 9–12 (35S) and 14–17 (45S) that correspond to the 35S U5 snRNPs and the mixture of B* and C complexes, respectively (Fig 3). To demonstrate the specificity of precipitations the antibodies were also pre-blocked with antigenic peptide. Following precipitation, the RNA was extracted, labelled with ^32^P-pCp, analysed by PAGE and visualised by autography. Fig 4 shows that the DDX35 antibody efficiently and specifically precipitated the U5 snRNA from the 35S fractions (lanes 3 and 4). A small amount of U5 snRNA was also precipitated from the 45S region (lanes 1 and 2) which most likely corresponds to the C complex present in these fractions. Some amount of the 5.8S and 5S ribosomal RNA (rRNA) was also present in precipitate but these appear to be non-specific. Thus, our results support the previously proposed idea that 35S U5 snRNPs are generated during splicing and represent the dissociation product of the spliceosome.

## Conclusions

U5 snRNP contains major protein components of the spliceosome catalytic core, which are Prp8 protein and two regulatory proteins, the helicase Brr2 and GTPase Snu114 proteins. Three types of particles containing U5 snRNP have been purified and characterised from HeLa nuclear extract. These include 20S U5 snRNP, 25S U4/U6.U5 tri-snRNP, and 35S U5 snRNP. The 20S U5 snRNP associates with the 10S U4/U6 snRNP to form stable 25S U4/U6.U5 tri-snRNP complex [15], which is integrated into the spliceosome during transition from the A to B complex [16]. The 35S U5 snRNP is formed within the spliceosome due to the association of the PRP19/CDC5L complex during spliceosome activation. After the completion of both catalytic steps of splicing, the spliceosome disassembles and the 35S U5 snRNP is released as the dissociation product of the spliceosome. Next, the 35S U5 snRNP is likely converted into the 20S-like U5 particle. At present, it is not known what is required to release PRP19/CDC5L from the 35S particles and recycle U5 snRNP for the new rounds of splicing.
